# Supplementary figures and images for: An objective, automated and robust scoring using fluorescence optical imaging to evaluate changes in micro-vascularisation indicating early arthritis
Source: PLoS One. 2022 Sep 27;17(9):e0274593. doi: 10.1371/journal.pone.0274593 (PMC9514628; doi:10.1371/journal.pone.0274593)

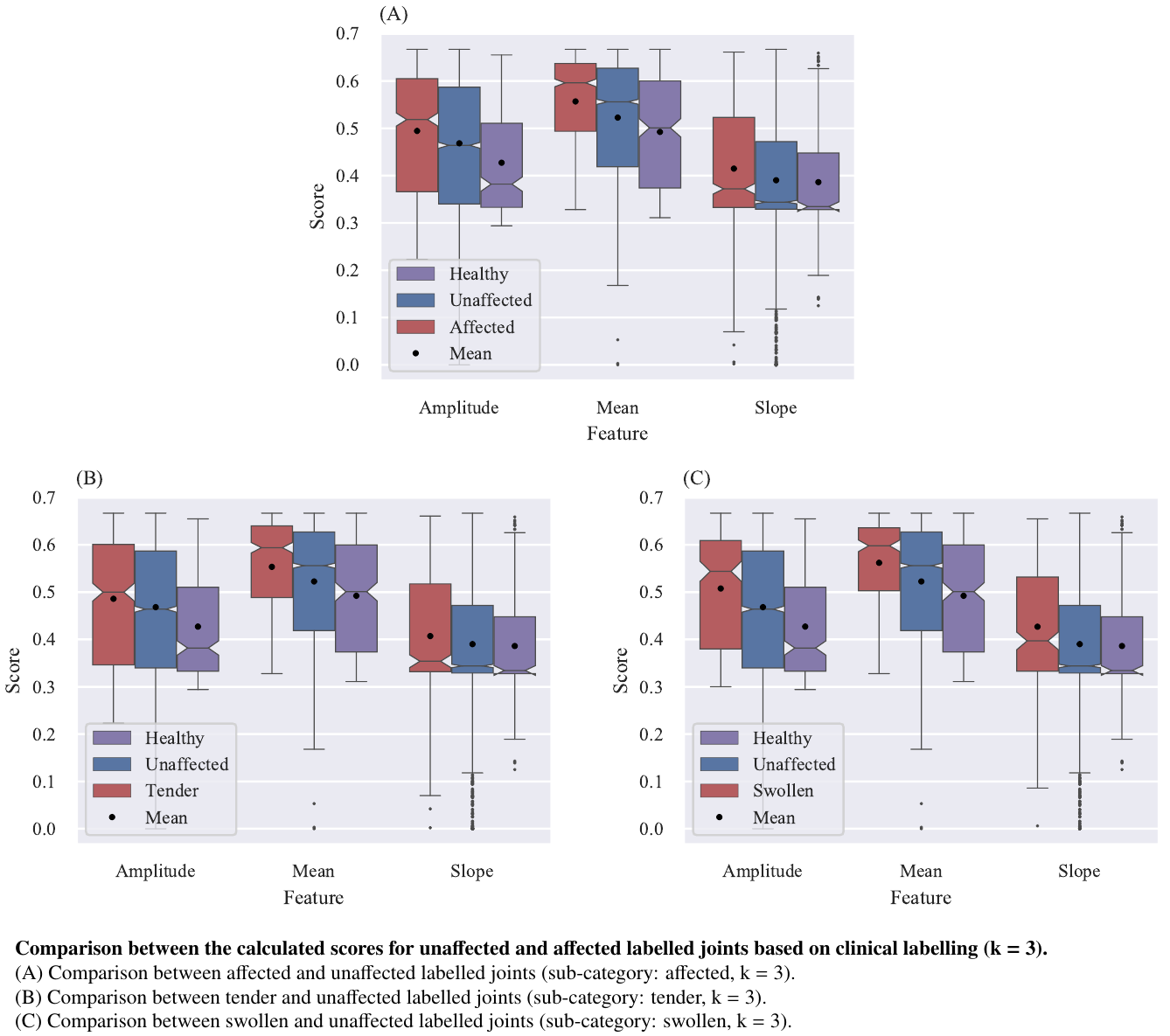

Supplement: S1 Fig — (A) Comparison between affected and unaffected labelled joints (sub-category: affected, k = 3). (B) Comparison between tender and unaffected labelled joints (sub-category: tender, k = 3). (C) Comparison between swollen and unaffected labelled joints (sub-category: swollen, k = 3). (TIF) [file pone.0274593.s001.tif]

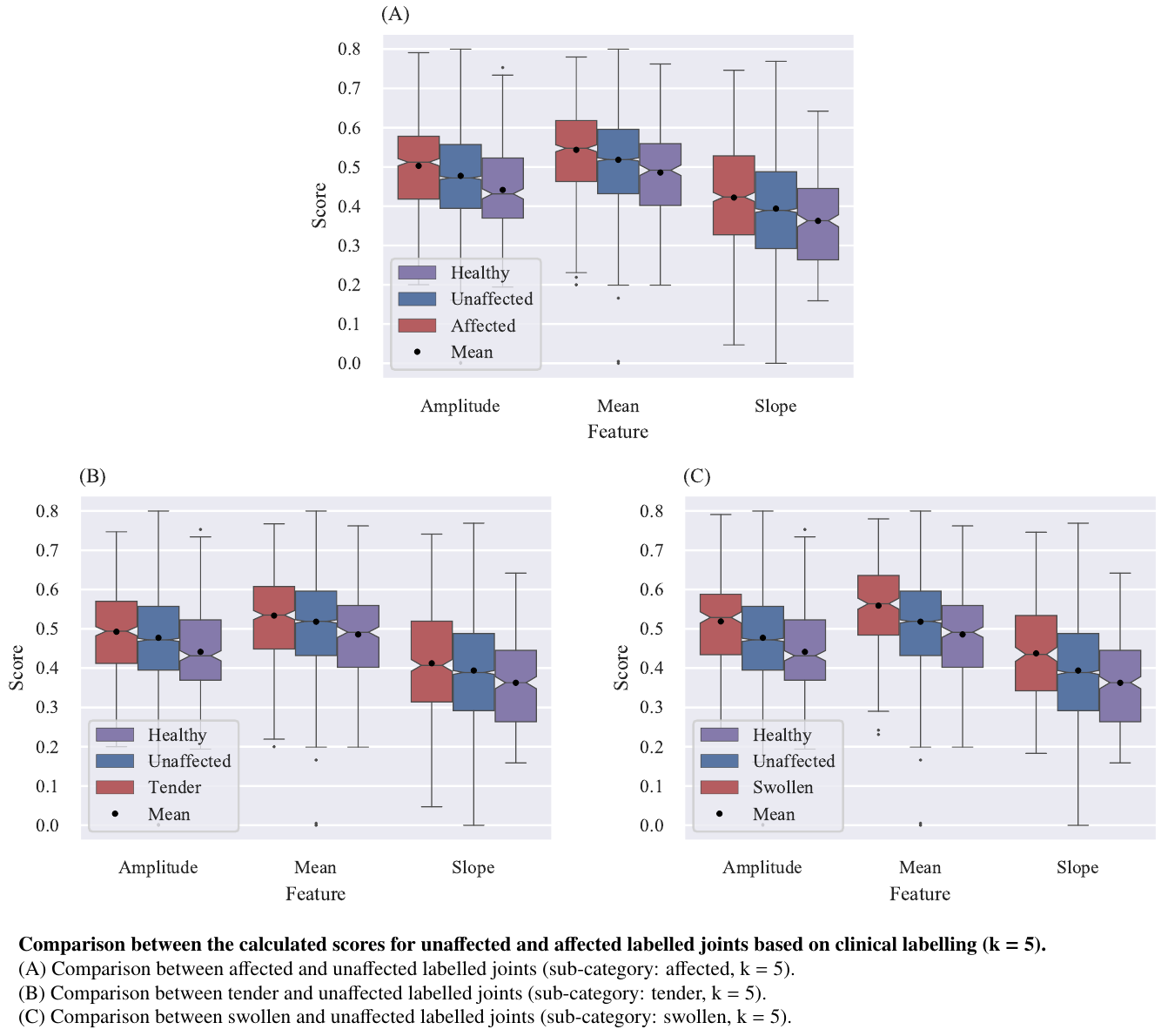

Supplement: S2 Fig — (A) Comparison between affected and unaffected labelled joints (sub-category: affected, k = 5). (B) Comparison between tender and unaffected labelled joints (sub-category: tender, k = 5). (C) Comparison between swollen and unaffected labelled joints (sub-category: swollen, k = 5). (TIF) [file pone.0274593.s002.tif]

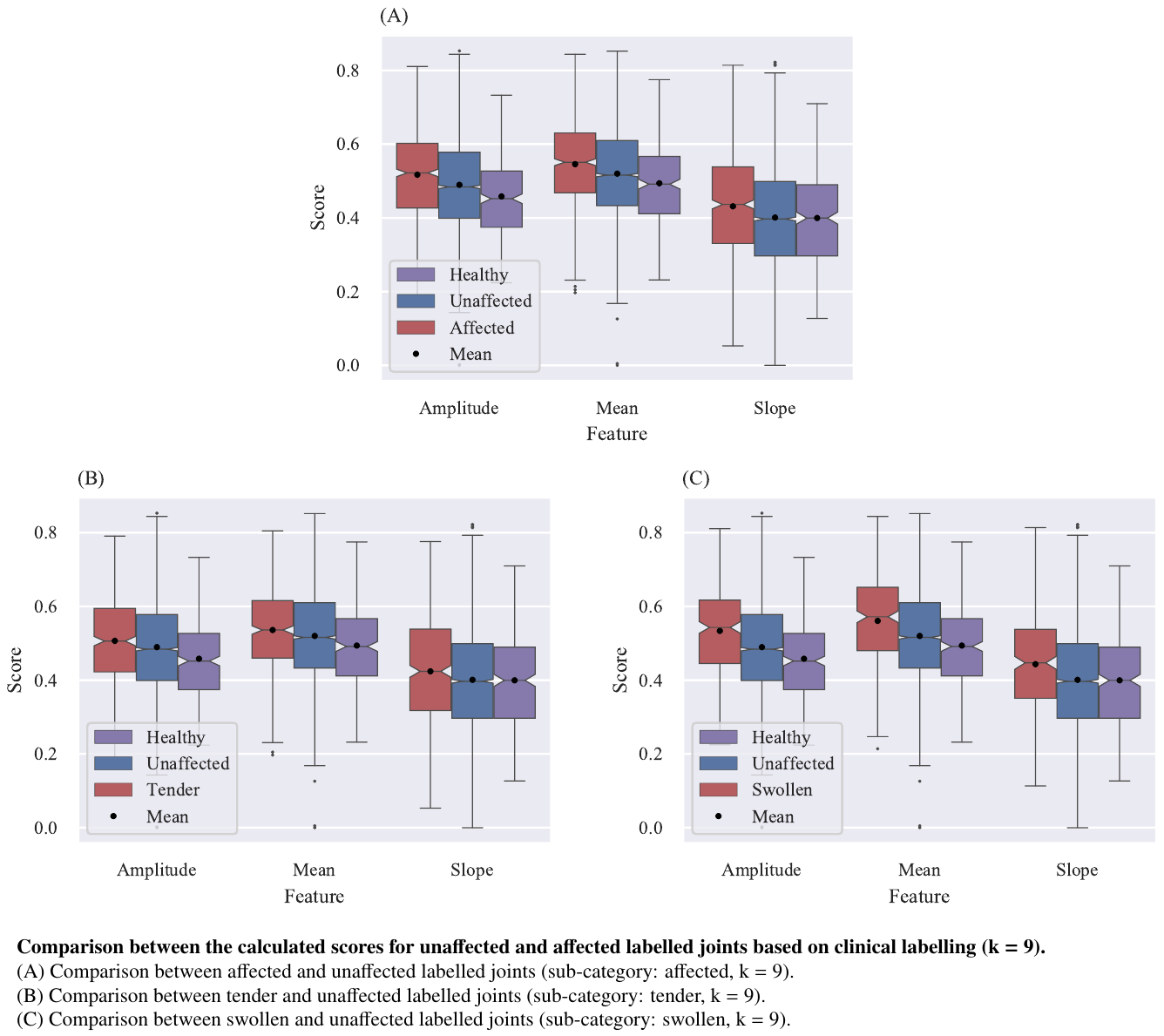

Supplement: S3 Fig — (A) Comparison between affected and unaffected labelled joints (sub-category: affected, k = 9). (B) Comparison between tender and unaffected labelled joints (sub-category: tender, k = 9). (C) Comparison between swollen and unaffected labelled joints (sub-category: swollen, k = 9). (TIF) [file pone.0274593.s003.tif]

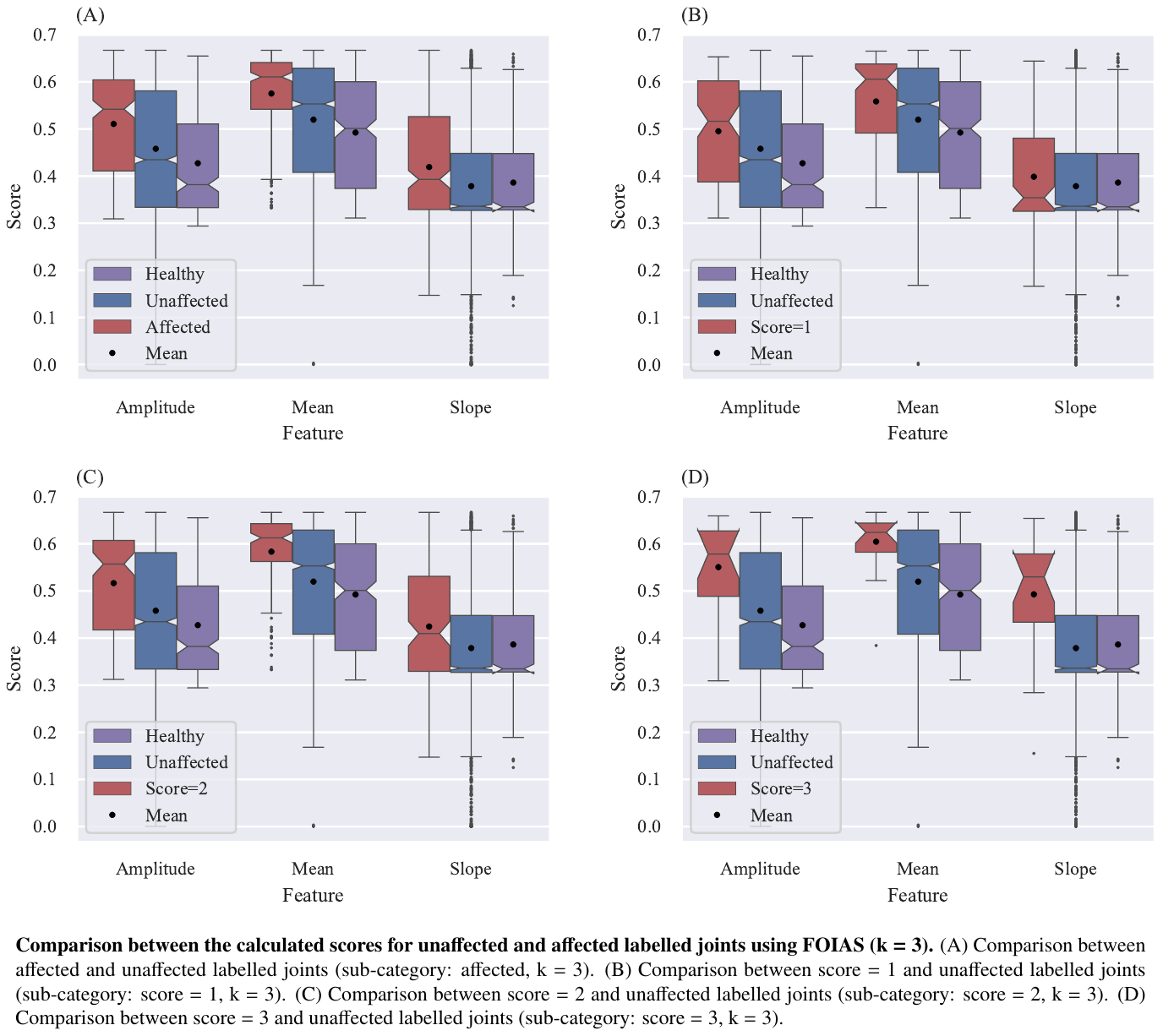

Supplement: S4 Fig — (A) Comparison between affected and unaffected labelled joints (sub-category: affected, k = 3). (B) Comparison between score = 1 and unaffected labelled joints (sub-category: score = 1, k = 3). (C) Comparison between score = 2 and unaffected labelled joints (sub-category: score = 2, k = 3). (D) Comparison between score = 3 and unaffected labelled joints (sub-category: score = 3, k = 3). (TIF) [file pone.0274593.s004.tif]

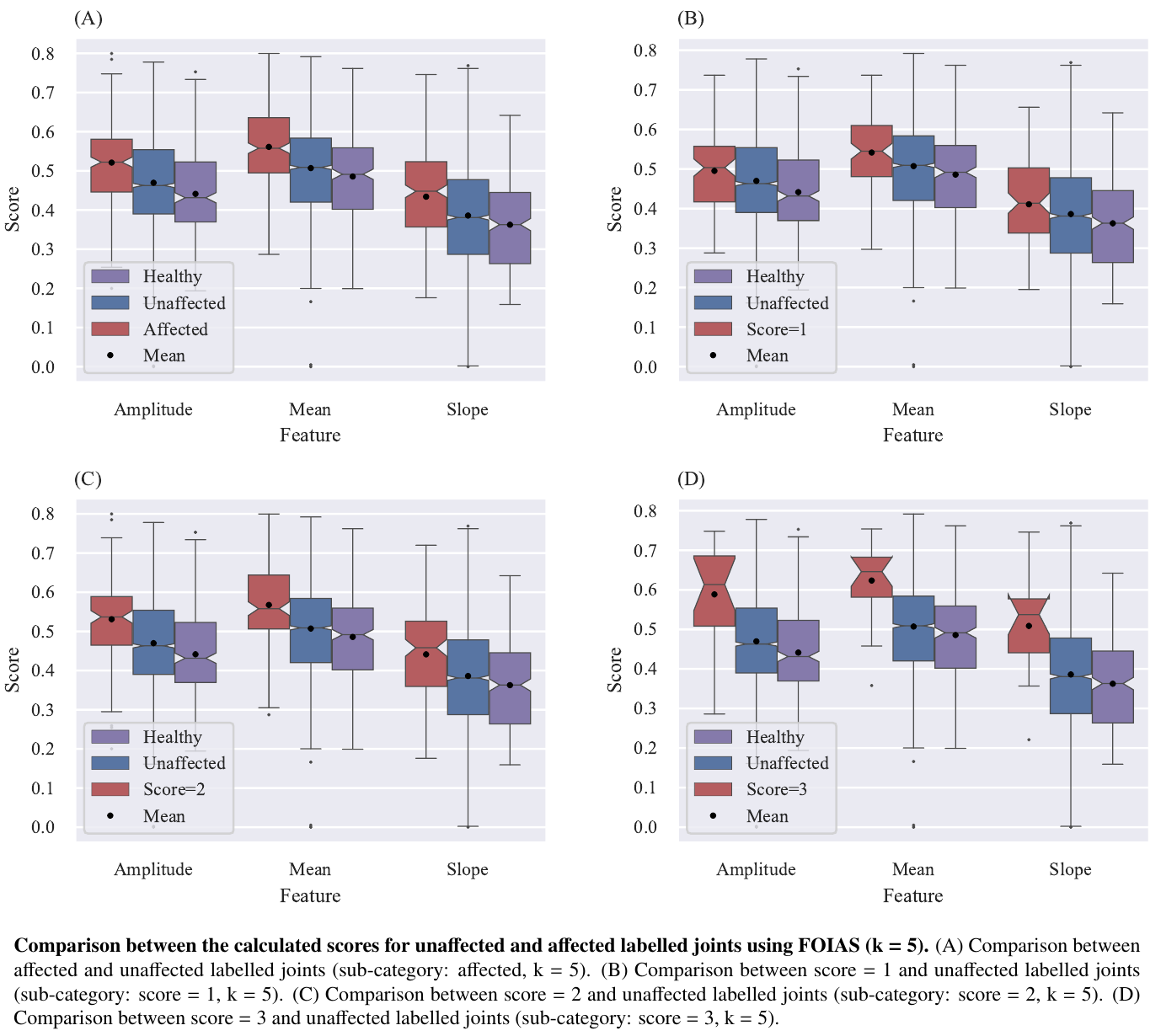

Supplement: S5 Fig — (A) Comparison between affected and unaffected labelled joints (sub-category: affected, k = 5). (B) Comparison between score = 1 and unaffected labelled joints (sub-category: score = 1, k = 5). (C) Comparison between score = 2 and unaffected labelled joints (sub-category: score = 2, k = 5). (D) Comparison between score = 3 and unaffected labelled joints (sub-category: score = 3, k = 5). (TIF) [file pone.0274593.s005.tif]

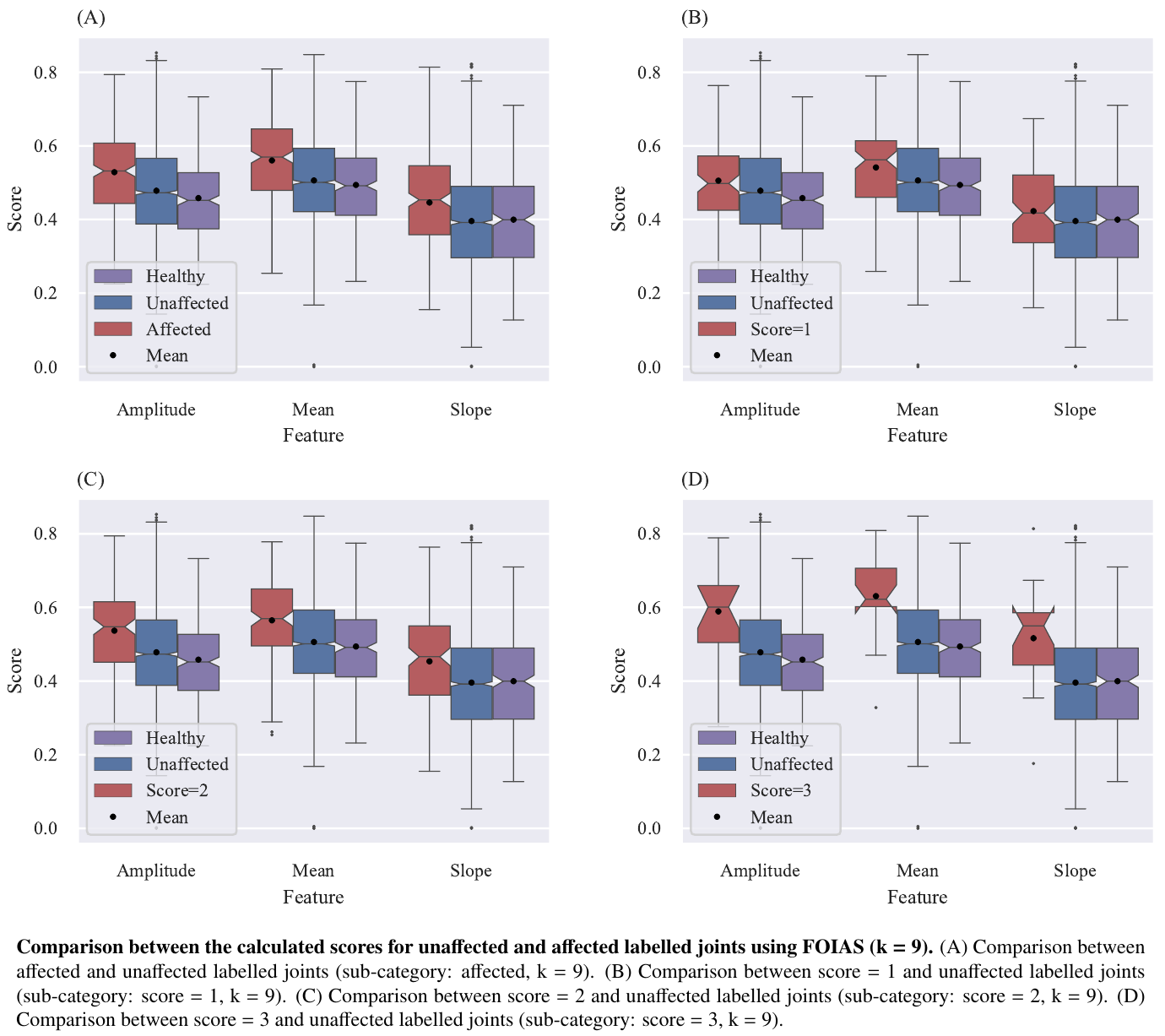

Supplement: S6 Fig — (A) Comparison between affected and unaffected labelled joints (sub-category: affected, k = 9). (B) Comparison between score = 1 and unaffected labelled joints (sub-category: score = 1, k = 9). (C) Comparison between score = 2 and unaffected labelled joints (sub-category: score = 2, k = 9). (D) Comparison between score = 3 and unaffected labelled joints (sub-category: score = 3, k = 9). (TIF) [file pone.0274593.s006.tif]
